# Allelic dropout in the endoglin (*ENG*) gene caused by common duplication beyond the primer binding site

**DOI:** 10.3389/fgene.2025.1571437

**Published:** 2025-06-11

**Authors:** Anna G. Shestak, Victoria A. Rumyantseva, Elena V. Zaklyazminskaya

**Affiliations:** ^1^ Medical Genetics Laboratory, Petrovsky National Research Center of Surgery, Moscow, Russia; ^2^ Research Center for Medical Genetics, Moscow, Russia

**Keywords:** allelic dropout, *ENG*, hereditary hemorrhagic telangiectasia, HHT, next-generation sequencing, Sanger sequencing, diagnostic yield, DNA diagnostics

## Abstract

Allelic dropout (ADO) is a common limitation of all PCR-based molecular diagnostic methods, leading to false-negative or false-positive results, depending on the allele that was dropped. We report a case of multiple locus-specific allele dropouts mediated by a common duplication beyond the primer-binding site of the endoglin *(ENG)* gene. We observed a family with hereditary hemorrhagic telangiectasia (HHT) where the HHT diagnosis in the proband (female, 71 years old) and two family members was based on the Curaçao criteria. A nonsense heterozygous c.831C>A (p.Y277*) mutation and a common homozygous duplication c.991+21_26dup in exon 7 of the *ENG* gene was revealed in the proband. Discrepancies were found between the obvious clinical HHT phenotypes of the two family members and the negative results of cascade familial screening based on capillary Sanger sequencing with classically designed oligoprimers. In addition, ADO was suspected due to the absence of c.991+21_26dup. We analyzed the primer-binding sites using gnomAD to reveal the cause of ADO. Amplicons with notable ADO were resequenced using alternative oligoprimers. Three primer pairs that were designed more distal (toward the 3′-end) after duplication were unable to amplify both alleles. Redesigning oligoprimers complementary to the narrow area successfully detected the heterozygous variant p.Y277* in two family members. The classical primer design for Sanger sequencing may lead to the inefficient amplification of exon 7 amplicons with duplications (up to 19% according to MAF in gnomAD). These results suggest that indels beyond the primer-binding sites may lead to allele loss and false-negative results in DNA diagnostics.

## 1 Introduction

Allelic dropout (ADO) is the selective amplification of alleles during polymerase chain reaction (PCR). ADO is a factor that limits the efficiency of DNA diagnostics using all PCR-based methods. The consequences of ADO are mostly loss of heterozygosity (false homozygosity) (Tester et al., 2006; Martins et al., 2011; Wang et al., 2012) or underrepresentation of alternative alleles in next-generation sequencing (NGS) data (Jeong et al., 2019; Shestak et al., 2021). Both factors affect fundamental and clinical genetic research.

The actual prevalence of locus-specific ADO and the lack of clinically relevant genetic variants have not been properly estimated. Presumably, ADO may affect up to 0.77% of the amplicons of the target gene panels (Shestak et al., 2021) with 14% of the variants per sample falling within that region (Zucca et al., 2016). The importance of this phenomenon is high given the scale of high-throughput sequencing in modern clinical practice.

Most cases of locus-specific ADO are caused by the presence of single nucleotide variants (SNVs) in primer-binding sites (Martins et al., 2011). The presence of differential allelic methylation and G-quadruplexes in some genomic regions and the simultaneous presence of both homopolymeric tracts and pseudogenes have been described as potential determinants of ADO (Stevens et al., 2017; De Cario et al., 2020).

Redesigning an alternative pair of primers is a standard step to confirm selective allele amplification. In most cases, this is an effective method for detecting molecular causes of ADO.

According to Blais et al. (2015), error rates vary significantly among different target amplification loci (Blais et al., 2015). Thus, it can be assumed that resequencing different target regions after redesigning primers for different loci does not always prevent this problem. However, the characteristics of the studied nucleotide sequences adjacent to the primers that influence the successful reduction of allele dropout events remain unknown. Moreover, it seems that the result obtained with only one pair of “validation” alternative primers will not always be sufficient to avoid ADO.

Hereditary hemorrhagic telangiectasia (HHT) is an autosomal dominant monogenic disorder, mostly familial with few *de novo* cases (Tørring et al., 2018). HHT associates with mutations in the endoglin (*ENG*) gene, also known as CD105 (39%–59% of cases), *ACVRL1* gene (25%–57% of cases), or *SMAD4* gene (1%–2%) in a subset of patients with HHT and juvenile polyposis (Viteri-Noël et al., 2022). Despite the diagnostic efficiency of approximately 90% for sequencing all known genes, a diagnosis cannot always be confirmed in individuals who are obligate carriers of a mutation.

The only case of false homozygosity of a splice site mutation c.817-3T>G (Variation ID: 3891317 in ClinVar) in the *ENG* gene was described in a woman with HHT due to ADO (Tørring et al., 2012). Sanger sequencing using an alternative pair of oligoprimers confirmed the true status of heterozygosity and the common duplication c.991+21_26dup (Variation ID: 213201 in ClinVar) as a hypothetical cause of allelic dropout.

In the present study, we demonstrated a case of multiple locus-specific ADO due to a non-primer-binding site and evaluated the possible contribution of ADO to genetic screening of the *ENG* gene. To our knowledge, this is the second reported case of an ADO of the *ENG* gene.

## 2 Materials and methods

**FIGURE 1:**
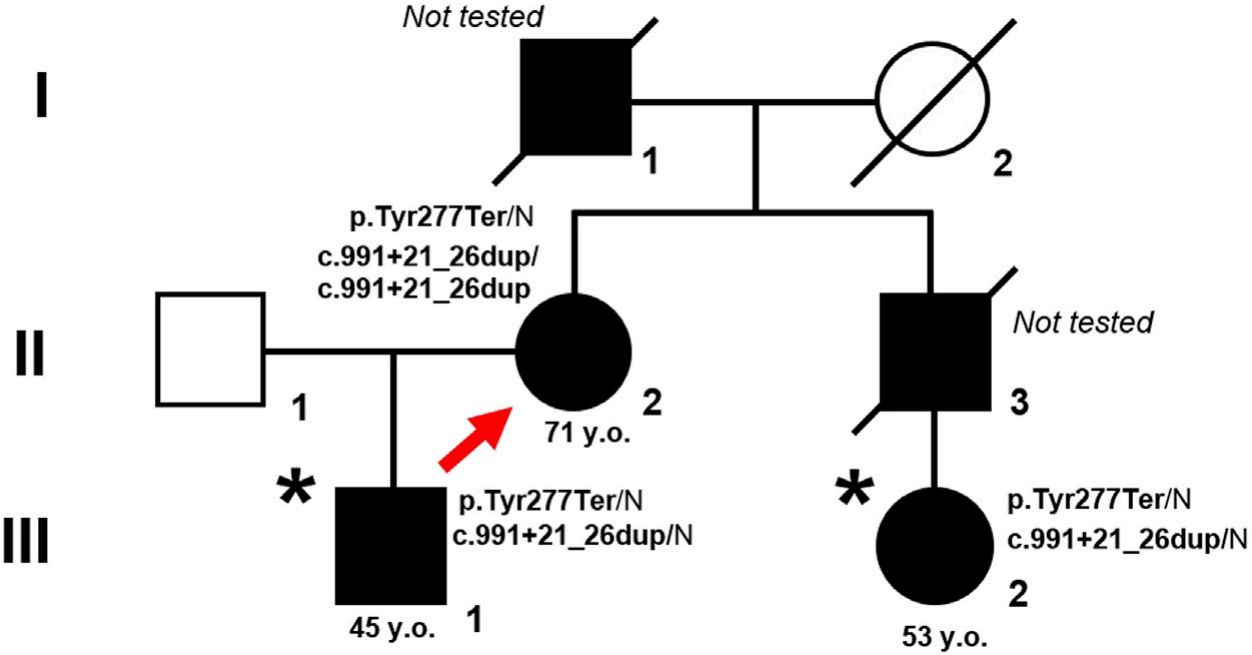
Pedigree of the family with HHT. Proband is marked by arrow. An asterisk indicates patients in whom the mutant allele was not initially detected due to ADO. Closed symbols represent affected family members, opened symbols represent healthy and non-tested family members.

HHT diagnoses were based on the family members: proband (II.2), son (III.1), and niece (III.2) based on the Curaçao criteria. The pedigree of the family is shown in Figure 1.

DNA was extracted from venous blood using a Quick-DNA Miniprep Plus Kit (Zymo Research Corp., Irvine, CA, USA) according to the manufacturer’s instructions. Direct capillary Sanger sequencing of the coding and adjacent regions of the *ENG* was performed on an ABI 3730XL DNA Analyzer according to the manufacturer’s instructions (Thermo Fisher Scientific, Waltham, MA, USA). The results of direct Sanger sequencing were visualized using Chromas software (Technelysium Pty Ltd., South Brisbane, Australia). The pathogenicity of the identified variants in the *ENG* gene were assessed according to ACMG (2015) guidelines (Richards et al., 2015).

Analysis of the forward and reverse primer binding sites along with the entire amplified region using the Genome Aggregation Database (gnomAD) v4.1.0 (Karczewski et al., 2020) was carried out to determine the cause of ADO. Alternative pairs of oligoprimers flanking the coding and adjacent intronic regions of exon 7 of *ENG* were designed using the open-source PerlPrimer software (Marshall, 2004) and NCBI Primer Blast. PCR protocol and annealing temperatures of the primers were optimized *in situ*.

Additionally, to ensure reproducibility of the study results, direct capillary Sanger sequencing of the amplicon from patient III.2 was performed in an alternative genetics laboratory.

Sequencing data from other patients tested using an AmpliSeq targeted gene panel consisting of the *ENG* gene (Thermo Fisher Scientific, Waltham, MA, USA) were analyzed to assess the representation of genetic variants in exon 7 and adjacent intronic areas. Oligoprimers were designed automatically using Ion AmpliSeq Designer^®^ (Thermo Fisher Scientific) containing 342 primer pairs for 14 genes (*CTGF, ENG, FBN1, FLNA, POSTN, RUNX2, SERPINE1, SMAD2, SMAD3, SMAD4, TGFB1, TGFBR1, TGFBR2, TGFBR3*), with a total length of 63 kb. Library preparation was performed using the Ion AmpliSeq Library Kit 2.0 according to the manufacturer’s instructions (Thermo Fisher Scientific, Waltham, MA, USA). Next-generation sequencing was performed using high-throughput semiconductor sequencing on an Ion PGM^TM^ System according to the manufacturer’s instructions (Thermo Fisher Scientific, Waltham, MA, USA). The average amplicon length in the panel was 235 bp, mean coverage with at least 20 reads- 95.2%, and the mean coverage with at least 100 reads-78.24%. Ion PGM^TM^ System data were processed with CoverageAnalysis and VariantCaller plugins available within the licensed Torrent Suite Software 5.6.0 and Ion Reporter Software (Thermo Fisher Scientific). NGS sequencing reads were visualized using the Integrative Genomic Viewer (IGV) tool (Robinson et al., 2011) with hg19 as the reference genome.

To exclude monoallelic amplification, amplicons with noted or suspected ADO cases were resequenced by direct Sanger sequencing using alternative non-overlapping oligoprimer pairs.

## 3 Results

**FIGURE 2:**
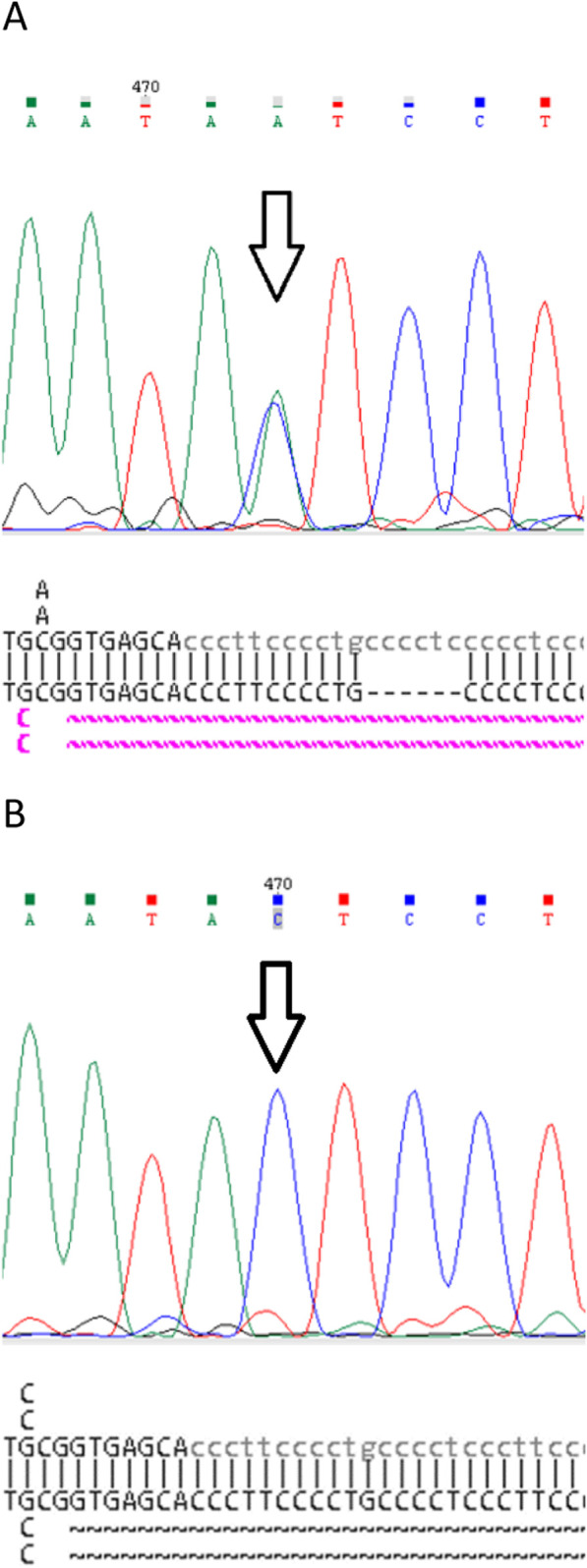
Fragments of the original Sanger sequencing chromatograms and NCBI BLAST reports of: **(A)** Proband. Pathogenic variant c.831C>A (p.Y277*) in heterozygous state (arrow) and common duplication c.991+21_26dup in homozygote state. **(B)** Family member III.2. Pathogenic variant c.831C>A (p.Y277*) and common duplication c.991+21_26dup are not detected.

Proband (female, 71 years old) with a clinical HHT diagnosis based on the Curaçao criteria was referred for genetic testing after genetic counseling. Direct Sanger sequencing of the coding region and adjacent areas of the *ENG* gene was performed. The rare genetic variant NM_001114753.3:c.831C>A (p.Y277*) (Variation ID: 579302 in ClinVar) of class IV pathogenicity (Likely Pathogenic) was identified in the proband (Figure 2A).

A search for the c.831C>A (p.Y277*) genetic variant in exon 7 of the *ENG* gene was carried out using direct Sanger sequencing in family members III.1 and III.2 showing clinical signs of HHT. However, this expected mutation was not found in either family member (Figures 1, 2B).

For a visual comparison of the chromatograms, proband files were used in which the mutation was clearly detected in the heterozygous state. In addition, when aligning the proband amplicon sequence to the reference genome using NCBI Blast (without using the default “low complexity” parameter), a homozygous duplication of 6 nucleotides, c.991+21_26dup, was identified in the 7th intron of the *ENG* gene. This duplication was recognized only when the parameter was used during alignment. We expected to detect a duplication in the amplicon sequences of patients III.1, and III.2; however, they were absent (Figures 1, 2B).

To verify the results obtained, we sequenced the amplicon of patient III.2 from the original pair of primers in another genetic laboratory. We suspected selective allele amplification in the amplicons of patients III.1 and III.2 because no duplication was detected in our and alternative laboratories.

This duplication occurs at a gnomAD v4.1.0 frequency of 19.23% in total and 18.09% in European (non-Finnish) populations (Date of access 20-01-2025) (Robinson et al., 2011), without altering DNA folding (Tørring et al., 2012). Tørring et al. (2012) identified the only genetic variant that was initially undetected in the proband’s original amplicon but was subsequently detected in a heterozygous state in all tested amplicons after using an alternative primer pair. Therefore, this intronic duplication between the reverse primer-binding site and the coding area served as a genetic variant marker to confirm selective allele amplification. A schematic of the study region is shown in Supplementary Figure S1.

To assess the presence of c.991+21_26dup in our patients, NGS sequences (targeted gene panel, the design of which includes the *ENG* gene) were analyzed for those patients who were sequenced for this targeted gene panel. We found a discrepancy in the detection/zygosity of this duplication in the 15 control DNA samples by comparing the results of exon 7 and adjacent intronic sequences obtained from the primers designed for NGS (targeted genes panel) and Sanger sequencing. The results of the PCR using oligoprimers subjected to ADO confirmed the monoallelic status of the amplicons in this group of patients (Supplementary Figure S2; Supplementary Figure S3).

Three primer pairs designed more distal (toward the 3′-end) after the expected duplication were unable to amplify both alleles (Table 1). UNAFold did not show any changes in DNA folding patterns due to duplication. We noticed that the region around the c.991+21_26dup duplication consisted of C and T nucleotides and formed dimeric complexes in the DNA double strand, which were much larger than the duplicated 6-nucleotide region (Supplementary Figure S4). Thus, three tested amplicons from each patient were exposed to ADO (Table 1). Re-design of the reverse oligoprimer ahead of c.991+21_26dup complementary to the narrow area within the coding sequence (total amplicon of 425 bp) of exon 7 and subsequent Sanger sequencing were only successful in detecting heterozygous c.831C>A (p.Y277*) genetic variants in the DNA fragments of patients III.1 and III.2. In consideration of variant segregation in ≥3 meioses (PP1 criterion), the p.Y277* variant was re-classified as Pathogenic (V) according to ACMG (2015) criteria. Characteristics of the oligonucleotide primers used for PCR and ADO analysis are presented in Table 2.

**TABLE 1:** Revealed ADO cases in Sanger sequencing results of the *ENG* gene.

Parameter	Tørring et al. (2012)	This study
Disease-causing variant	c.817-3T>G (Class V, Pathogenic)	p.Y277* (Class V, Pathogenic)
Location of the pathogenic variant	NM_001114753:intron 6	NM_001114753:exon 7
ADO-causing benign variant	c.991+21_26dup	c.991+21_26dup
MAF^a^	0.1809	0.1809
Size and genomic coordinates (hg19) of the amplicons subjects to ADO	334 bp, сhr9:130587331-130586998	741 bp, сhr9:130587740-130587000, 605 bp, сhr9:130587541-130586937, 915 bp, сhr9:130587541-130586626
Marker variant for ADO	c.817-3T>G^¥^	c.991+21_26dup*
Zygosity	Hetero	Hetero
Type of event	Missing of WT allele^¥^ (false homozygosity)	Missing of mutant allele (*false-negative)
Sequencing platform with ADO occured	Sanger sequencing	Sanger sequencing
Re-sequencing platform	Sanger sequencing	Sanger sequencing
Size and genomic coordinates (hg19) of amplicons without ADO	929 bp, сhr9:130587544-130586616	425 bp, сhr9:130587541-130587116
Number of patients with confirmed ADO	1	2

^a^ MAF in European (non-Finnish) population based on gnomAD v4.1.0 data.

¥, * indicate the link between the type of event and the variant marked.

**TABLE 2:** Oligonucleotide primers used for PCR and ADO analysis of the amplicons of the *ENG* gene. The primer-binding sites of the new primers with proven efficacy against ADO in this study are shown in bold and underlined.

Analyzed genetic region	Size of the amplicon, bp	Genomic coordinates of the amplicon (hg19)	Forward primer sequence	Primer length	Annealing T (°C)	Reverse primer sequence	Primer length	Annealing T (°C)	ADO occured	Reference
Ex 6-7	741	сhr9:130587740-130587000	5′-CAC​CTG​GCC​AGG​TAA​GAG​T-3′	19	61	5′-CTG​TTG​GTG​AAG​CAC​CTC​TGT-3′	21	63	Yes	This study
Ex 7	605	сhr9:130587541-130586937	5′-TCA​TCG​ACG​CCA​ACC​ACA​ACA-3′	21	65	5′-GTT​CCC​ATG​TGC​AGA​TGA​G-3′	19	59	Yes	This study
Ex 7	915	сhr9:130587541-130586626	5′-TCA​TCG​ACG​CCA​ACC​ACA​ACA-3′	21	65	5′-GCA​CAC​TTT​GTC​TGG​ATC​AAG-3′	21	60	Yes	This study
Ex 7	425	сhr9:130587541-130587116	5′-**TCA​TCG​ACG​CCA​ACC​ACA​ACA-3′**	21	65	5′-**CGG​TAG​CTC​CAC​GAA​GGA​TG-3′**	20	63	No	This study
Ex 7	334	сhr9:130587331-130586998	5′-CTG​GCA​TAA​CCC​TGG​CTG-3′	18	61	5′-GTT​GGT​GAA​GCA​CCT​CTG​TGT-3′	21	63	Yes	Tørring et al. (2012)
Ex 7	929	сhr9:130587544-130586616	5′-GGC​TCA​TCG​ACG​CCA​ACC​ACA​ACA-3′	24	70	5′-ACA​AAG​TGT​GCC​GAC​GAC​GCC-3′	21	69	No	Tørring et al. (2012)

We believe that these multiple ADO events were caused by the common intronic variant c.991+21_26dup, located at the non-primer-binding site. This duplication affected all amplicons that were located. Selective allele amplification was confirmed in all the cases studied.

## 4 Discussion

ADO is a common and underestimated phenomenon affecting accuracy of genetic results. Due to different mechanisms, a single allele is amplified exclusively or predominantly, leading to the overrepresentation of homozygosity (Wang et al., 2012). ADO can often be suspected based on the false homozygosity of the variant of interest, in which the zygotic state contradicts the clinical concept. In addition, for dominant mutations, the primary concern of ADO is a false-negative result caused by the amplification failure of a mutant allele (Mullins et al., 2007).

Tørring et al. (2012) described a case of false homozygosity of the splice site mutation c.817-3T>G in intron 6 of the *ENG* gene in a woman with HTT. The results were reproduced using DNA re-isolated from an independent portion of blood. This disease has an autosomal dominant inheritance pattern, and the presence of biallelic mutations usually leading to lethality *in utero*. Additionally, the patient’s daughter was clinically healthy, indicating a possible diagnostic error. After analyzing the haplotypes for the proband and family members, the authors found no signs of recombination or crossing-over (Tørring et al., 2012). Sanger sequencing using an alternative primer pair revealed true heterozygosity. However, no SNV was detected in the original primer-binding sites; the only genetic variant potentially significant for ADO found in this family was a common duplication of 6 nucleotides (c.991+21_26dupCCTCCC) between the reverse binding site and a coding region of exon 7 (Tørring et al., 2012). Bioinformatic analysis showed that this duplication does not change DNA folding significantly. However, in a cohort of 37 carriers it was visualized only when sequencing was carried out using the new primers, indicating ADO using the original pair of primers (Tørring et al., 2012).

To our knowledge, this is the first report of ADO due to a common genetic variant leading to incorrect genotyping of the *ENG* gene. This is also one of the few reports describing the cause of ADO located in a non-primer binding site.

The case of ADO described by Tørring et al. (2012) is an example of an obvious discrepancy between genotype and phenotype due to detected false homozygosity for the c.817-3T>G mutation. Interestingly, the patient had a splicing mutation (c.817-3T>G), and a common duplication, (c.991+21_26dup; minor allele frequency (MAF) 0.1923 in gnomAD v4.1.0), the cause of ADO, located on different alleles (trans-position).

We present a case of a nonsense heterozygous mutation p.Y277* and a common homozygous duplication c.991+21_26dup in the *ENG* gene identified in exon 7 in our proband but absent in the DNA of the family members. We suspected selective allele amplification in the amplicons of patients III.1, and III.2 due to undetected duplication. After reselecting a pair of alternative primers similar to the new primers published by Tørring et al. (2012) we did not see any duplication again, probably due to the suspected monoallelic state of our amplicons. In total, in the three sequenced amplicons of *ENG* (Table 1) with expected heterozygous duplication, we observed results from only one wild type (WT) allele of two, without genetic substitutions.

Subsequent sequencing using an alternative pair of primers for a short amplicon (425 bp) ahead of c.991+21_26dup allowed us to successfully identify the heterozygous mutation p.Y277* in family members III.1 and III.2.

Notably, the relatives of the proband analyzed in our study have both mutation and duplication located on 1 allele (*cis*-position), in contradistinction to the clinical case described by Tørring et al. (2012).

We observed that the region consisting of C and T nucleotides flanking the c.991+21_26dup duplication was much larger than the duplicated 6-nucleotide region implied by the nomenclature. The Gibbs free energy (ΔG) for the formation of dimers of this sequence at 37 °C was −17.44 kcal/mol which decreases to −8.54 kcal/mol at 60°C, as verified by open-source software PerlPrimer (Marshall, 2004) (Supplementary Figure S4) and OligoAnalyzer™ Tool. The formation of stable dimeric complexes as well as hairpins, especially at the 3′end, can create an alternative landing site for polymerase.

We believe that this non-primer binding site and such a sequence within the amplicon, will directly affect the stability of the Taq polymerase binding site. Thus, the direct genetic cause of ADO may be much larger than the original description by Tørring et al. (2012).

We also found that amplicons obtained from the same forward sequence but different reverse sequences resulted in the amplification of one or two alleles. In other words, the duplication affects only one primer-binding site, specifically the reverse. The distance in nucleotides between the duplication and our ADO-sensitive reverse primers (excluding the number of nucleotides in them) amounted to 32, 97, and 406 nucleotides, respectively, indicating that the influence of duplication on the studied region of the amplicon may be extensive (Tables 1, 2).

It would be interesting to evaluate whether the presence of SNV(s) in the non-primer binding site affects PCR. Lam and Mak (2013) described a case of ADO caused by a non-primer binding site SNV in the *FAH* gene due to strong secondary hairpin structure formation in PCR products, leading to amplification failure. The authors found that ADO from the original primer pair was reproduced many times by changing the PCR conditions: primer annealing temperatures, different magnesium concentrations, and different PCR kits with different polymerase enzymes (Lam and Mak, 2013). This provides evidence of the dependence of ADO on the nucleotide sequence, but not on the PCR conditions.

When ADO is suspected, amplicons generated using alternative primers should be tested first. To determine the cause of ADO, it is necessary to check the original primer-binding sites using an online resource (gnomAD). Hence, sequence variants outside the primer-binding sites cannot be suspected when checking the primer regions using validation resources.

In our case, the presence of c.991+21_26dup in all three sequenced amplicons caused ADO. Using the 4th pair of primers for a short amplicon without the c.991+21_26dup mutation, we identified the true heterozygous state of p. Y277* mutation (Table 2).

We suspect that one alternative pair of primer is not always sufficient to avoid all types of ADO and confirm the true allelic status of the studied amplicons.

This could be a dangerous pitfall of misdiagnosis if sequencing from additional alternative primer pair(s) or from a different sequencing platform is not performed in cases where ADO is suspected.

When ADO is suspected, it is critical to link genetic data with clinical findings and family history to minimize potential DNA diagnostic errors due to false-negative or false-positive results.

False homozygosity (false-positive result) is the most common consequence of ADO. This should be confirmed with parental genotype analysis whenever possible and differentiated from true homozygosity in cases of consanguinity in the family or copy number variations.

In contrast, a false-negative result due to an ADO is an event that can rarely be tested for potential ADO. Therefore, the risk of such events may be greater than normal.

In these cases, the results do not correlate with the clinical findings, raising a question about the actual cost-effectiveness of an approach to continue comprehensive genetic testing, searching further for genetic variants instead of checking for ADO.

Thus, there are few studies on the types of ADO caused by a variant localized beyond the primer binding site (Tørring et al., 2012; Lam and Mak, 2013). In these studies, ADO was suspected because of false-positive DNA diagnostic results that did not imply true homozygosity, true hemizygosity, or consanguinity of the patients.

The point ADO causes in non-primer binding site had been reported by Lam and Mak in 2013 (Lam and Mak, 2013). The ADO from the original primer pair was reproduced many times when the PCR conditions were changed. The allele in *cis* with NM_000137.1(FAH):c.961-35C (rs2043691) formed a stronger hairpin structure, leading to amplification failure of the maternal wild-type allele and apparent homozygosity of the paternal deletion NM_000137.1(FAH):c.1035_1037del in the proband (Lam and Mak, 2013).

Contrastingly, in our study, the cause of ADO is not point, but extensive, in which the affected allele is the one that drops out.

However, our work and the Lam and Mak study converge on the same phenomenon: the location of ADO is, in *cis*-position to all genetic variants of the affected allele.

Therefore, the *cis*-position of ADO may be an additional risk factor, which can result in either a false-positive or false-negative outcome. In case of a false-positive diagnostic result, ADO may more likely be under suspension by investigators than a false-negative result. Thus, the scale of the problem in *cis*-type ADO may be enormous, hiding a significant percentage of genetic variants that are otherwise invisible to researchers.

It is important to note that locus-specific allelic dropout can occur in a sample of the relative but not the proband, which can be explained by the characteristics of the locus and the zygotic status of the variants present in it.

With standard primer designs for Sanger sequencing, exon 7 amplicons with duplications (up to 19% according to MAF) can be amplified inefficiently, which might significantly reduce the DNA diagnostic yield for HHT patients. Other laboratories may identify ADO in this region of the *ENG* gene as well.

Previously, we have demonstrated that ADO is a common phenomenon in both NGS and Sanger sequencing results (Shestak et al., 2021). We estimated that oligoprimer design without ADO data affects the amplification efficiency up to 0.77% of targeted gene panels amplicons (Shestak et al., 2021).

The actual ADO rate might depend on the number of oligoprimer pairs. However, ADO-causing SNV in non-primer binding sites are more difficult to identify than SNVs in primer binding sites. Checking for SNV(s) exclusively at primer-binding sites during the primer design process is insufficient to avoid all types of allelic dropouts. Therefore, it was necessary to check the entire amplified region. Non-primer-mediated ADO may contribute significantly to the unforeseen loss of genetic data in DNA diagnostics. It would be interesting to analyze the genomic sequences for further identification of such regions within the studied amplicons and their influence on the selective amplification of alleles.

In summary, we report a case of multiple locus-specific allele dropouts mediated by a common duplication located in a non-primer-binding site of the *ENG* gene. When designing oligoprimers, we propose to check not only single nucleotide variations in primer binding sites but also indels within the studied amplicons to avoid selective allele amplification and loss of data in DNA diagnostics. The true prevalence of ADO remains unknown; however, we assume that it may be much higher than expected. An algorithm that analyzes template sequences by considering indels located within all amplification regions would be useful.

## Data Availability

The original contributions presented in the study are publicly available in NCBI using accession number SCV002575091.2.
