# Supplementary material for: Allelic dropout in the endoglin (ENG) gene caused by common duplication beyond the primer binding site
Source: Front Genet. 2025 Jun 11;16:1571437. doi: 10.3389/fgene.2025.1571437 (PMC12261672; doi:10.3389/fgene.2025.1571437)

Supplementary Figure 4. *In-silico* assessment of the 36-nucleotide genomic region containing the c.991+21_26dup in the *ENG* gene by PerlPrimer v1.1.21. Formation of dimers in the DNA double strand at 37 °C (A) and at 60 °C (B).

A

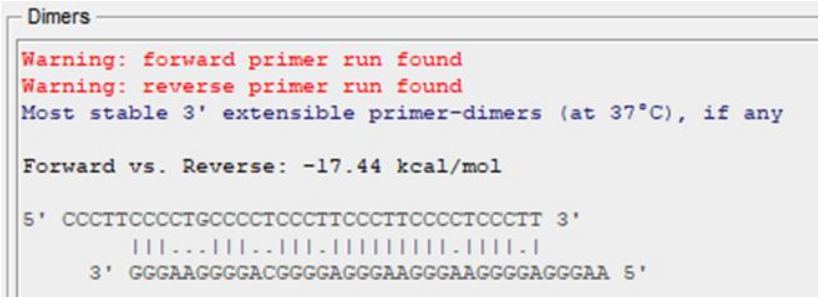

B

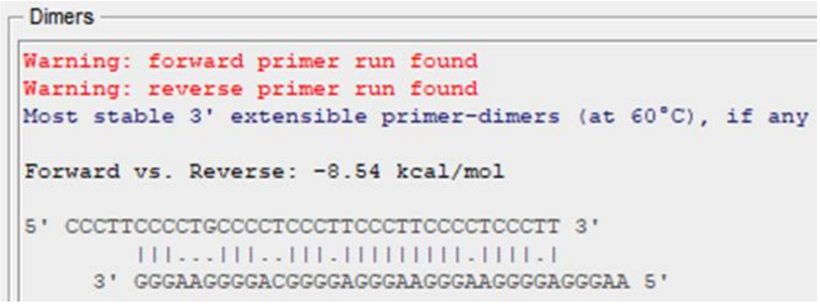

Supplement: Supplementary file 1 [file Image4.pdf]
