# Supplementary material for: Allelic dropout in the endoglin (ENG) gene caused by common duplication beyond the primer binding site
Source: Front Genet. 2025 Jun 11;16:1571437. doi: 10.3389/fgene.2025.1571437 (PMC12261672; doi:10.3389/fgene.2025.1571437)

Supplementary Figure 2. Discrepant results of genetic testing of exon 7 of *ENG* gene due to allelic dropout:

A. Ion Torrent PGM sequencing results using AmpliSeq primers. A common duplication c.991+21_26dup to be heterozygous in patient CTD637.

B. Ion Torrent PGM sequencing results of amplicon libraries of patient CTD637 using primers, alternative to AmpliSeq. A common duplication c.991+21_26dup is not detected due to ADO.

A

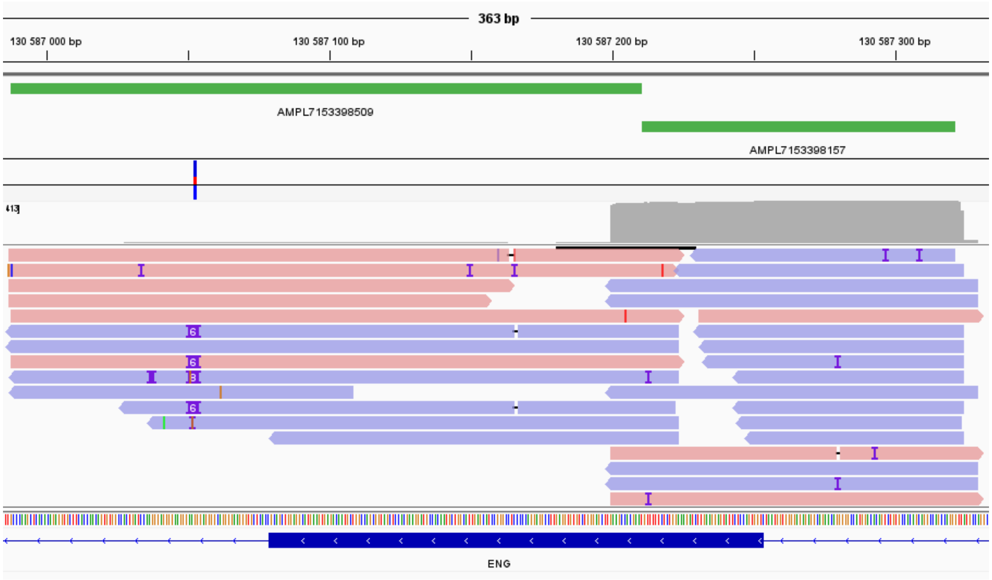

B

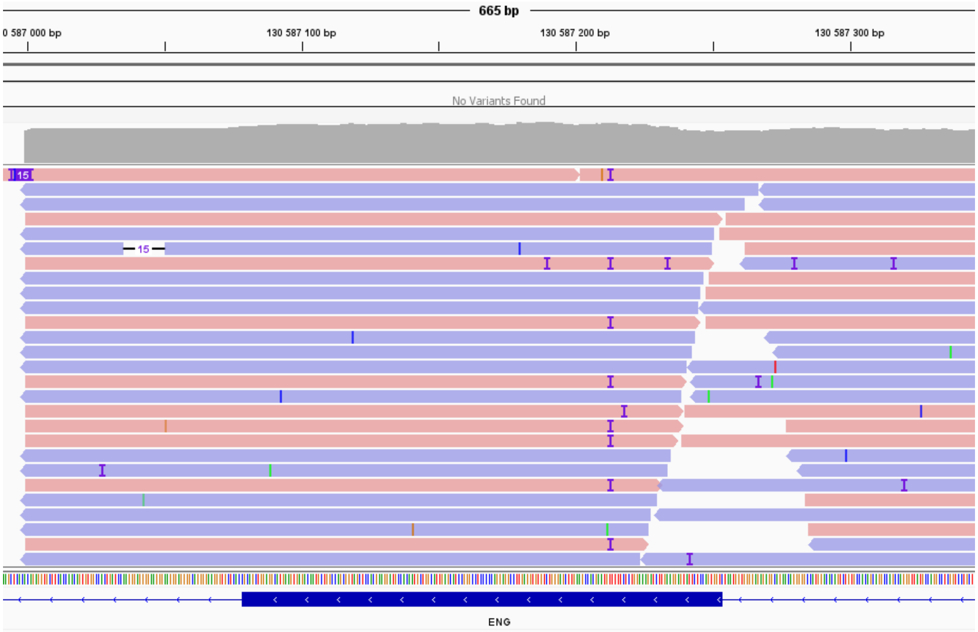

Supplement: Supplementary file 2 [file Image2.pdf]
