# Supplementary material for: Allelic dropout in the endoglin (ENG) gene caused by common duplication beyond the primer binding site
Source: Front Genet. 2025 Jun 11;16:1571437. doi: 10.3389/fgene.2025.1571437 (PMC12261672; doi:10.3389/fgene.2025.1571437)

Supplementary Figure 3. Picture of a gel electrophoresis using 3% agarose gel **(A)** and 8% polyacrylamide gel **(B)** of amplicons carrying heterozygous c.991+21_26dup obtained from ADO-susceptible primers of NGS-sequenced patients. Confirmation of the monoallelic status of the studied amplicons.

LAD - the molecular marker (ladder)

bp – base pairs

A

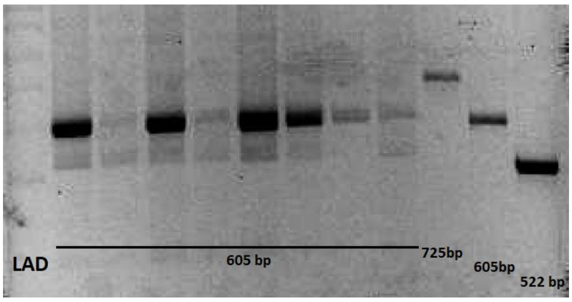

B

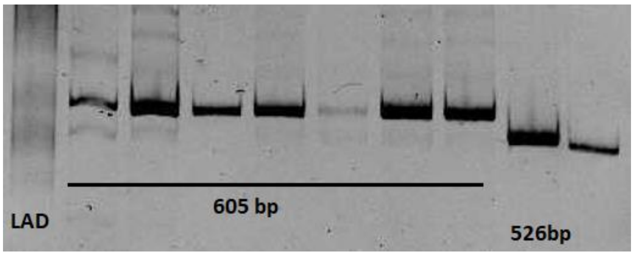

Supplement: Supplementary file 3 [file Image3.pdf]
