# Supplementary figures and images for: Allelic dropout in the endoglin (ENG) gene caused by common duplication beyond the primer binding site
Source: Front Genet. 2025 Jun 11;16:1571437. doi: 10.3389/fgene.2025.1571437 (PMC12261672; doi:10.3389/fgene.2025.1571437)

**Supplementary Figure 1.** Schematic representation of the region studied

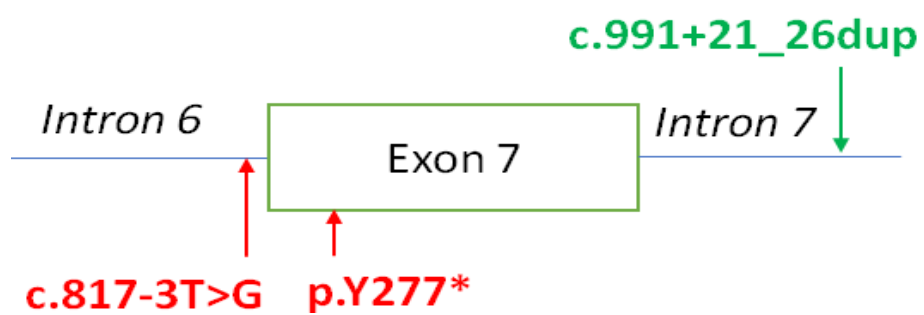

Supplement: Supplementary file 4 [file Image1.pdf]
